# Bottled Water: United States Consumers and Their Perceptions of Water Quality

**DOI:** 10.3390/ijerph8020565

**Published:** 2011-02-21

**Authors:** Zhihua Hu, Lois Wright Morton, Robert L. Mahler

**Affiliations:** 1 Department of Sociology, Iowa State University, 103 East Hall, Ames, IA 50011, USA; E-Mail: lwmorton@iastate.edu; 2 PSES Department, University of Idaho, P.O. Box 442339, Moscow, ID 83844, USA; E-Mail: BMAHLER@uidaho.edu

**Keywords:** bottled water, water quality perceptions, ground water quality

## Abstract

Consumption of bottled water is increasing worldwide. Prior research shows many consumers believe bottled water is convenient and has better taste than tap water, despite reports of a number of water quality incidents with bottled water. The authors explore the demographic and social factors associated with bottled water users in the U.S. and the relationship between bottled water use and perceptions of the quality of local water supply. They find that U.S. consumers are more likely to report bottled water as their primary drinking water source when they perceive that drinking water is not safe. Furthermore, those who give lower ratings to the quality of their ground water are more likely to regularly purchase bottle water for drinking and use bottle water as their primary drinking water source.

## 1. Introduction

Consumption of bottled water is increasing by ten percent every year worldwide, with the fastest growth seen in the developing countries of Asia and South America [1]. The United States (U.S.) is the largest consumer market for bottled water in the world. The U.S. consumption of bottled water in 2008 was estimated to be 8.6 billion gallons, or 27.6 gallons per person [2]. Despite the common belief that bottled water is safer to drink and has better taste than tap water, scientific studies have shown that the belief is not necessarily true [3,4]. Research also shows that the sales and consumption of bottled water can have environmental and social impacts whose consequences are yet to be fully understood [5–7]. After years of substantial growth in sales, the U.S. bottled water market is recently slowing down. The current economic downturn may have played a part in the drop; however, environmental concern is also an important factor. Some research has found that environmental awareness campaigns may have curbed consumer demand [8–10].

Previous studies about bottled water have focused on its production, regulation, sales and consumption, and criticism and concerns. However, few researchers have examined the relationship between consumer use of bottled water and perceptions of drinking water quality. In this article, the authors explore the demographic and social factors associated with bottled water users in the U.S. and the relationship between bottled water use and perceptions of the quality of local water supply. A brief discussion of bottled water and tap water and bottled water consumers is used to develop several hypotheses. These hypotheses are tested using a national dataset representing twenty-one U.S. states. Results and discussion are followed by implications directed toward educators and public policy makers as they fund and develop programs that promote knowledge about health and local drinking water.

### 1.1. Bottled Water vs. Tap Water

Bottled water has been used in place of tap water for its convenience, better taste, and perceived purity [1,3,11]. Perceptions of bottled water being of higher quality, however, are challenged by the increasing number of water quality incidents with bottled water [12]. A study showed that only five percent of the bottled water purchased in Cleveland, Ohio had the required fluoride recommended by the state, whereas the sampled tap water 100% met this requirement [3]. The same experiment also conducted bacteria count on both bottled water and tap water samples. The result showed that all of the tap water samples had a bacterial content under 3 CFUs/mL (colony-forming unit, a measure of viable bacterial or fungal numbers) and the bottled water samples' bacterial content ranged from 0.01–4,900 CFUs/mL. Although most of the water bottle samples were under 1 CFU/mL, there were 15 water bottle samples containing 6–4,900 CFUs/mL [3]. Another study focusing on the temperature and duration of storage for bottle water found that the bacterial growth in bottled water was markedly higher than that in tap water, especially at higher temperatures [4].

Many scientific reports on bottled water urge increased public awareness and development of guidelines/regulations on the industry of bottled water [1]. Incidents with bottled water quality are largely reported as associated with lenient regulations on bottled water. Bottled water plants are subject to the U.S. Food and Drug Administration (FDA) monitoring and inspection. Despite specific inspection requirements, bottled water plants are given low priority for safety inspection compared with other food plants because of FDA’s staffing and financial constraints [13]. The “Nutrition Facts” label on bottled water usually shows only limited information about the water [1].

Despite the popularity of bottled water in the U.S., there are a number of environmental and social concerns. Plastic bottles are a waste problem adding to landfill overload when not recycled. Water bottling plants have impacts on local groundwater aquifers and streams [5]. Taking too much water can reduce or deplete groundwater reserves and reduce the flow of streams and lakes, causing stress on ecosystems. Although 75% of the world bottled water is produced and distributed on a regional scale, trading and transporting the other 25% bottled water also raises the concern for pollution and carbon dioxide emission [6]. The price of bottled water is on average 500 to 1,000 times higher than that of tap water [6], contributing to concern for affordable access to drinking water. Limited resource populations that use bottled water for drinking are least able to afford the high cost associated with bottled water [1]. Another issue associated with increased consumption of bottled water is that it can erode public tap water revenues and the capacity of governments to provide necessary improvements in basic water infrastructure [7].

### 1.2. Consumers of Bottled Water

Eighty-five million bottles of water are consumed in the United States every day and more than thirty billion bottles a year [14]. The adoption of a health preventive action like drinking bottled water is suggested to be influenced by perception of risk associated with drinking water [15]. The perception of risk is also thought to be closely related to the subjective assessment of drinking water quality [11]. This suggests that perceptions of drinking water safety and beliefs about the ground and surface water quality in a local area might be explanatory factors for a decision to select bottled water over tap water.

Another safety factor influencing consumer decision to select bottled water over tap water is the type of water supply system where the consumer lives. Small water systems (small town, tribal system, rural water district) [16] in the U.S. were found to have problems complying with federal/state quality standards. According to one study, due to inadequate funding and facilities, small water systems reportedly violated federal drinking water regulations more frequently than larger ones [11]. Although the number of public water consumers whose water does not meet current standards has decreased significantly over years, the task of water regulation is still challenging given both the financial limitations and increasing public concern about their drinking water [11].

Socio-economic status is also a factor affecting consumer decisions, particularly given the high cost associated with bottled water. Gender and education differences have been found to affect preference of bottled water over tap water because of their noted differences in perception of environmental risk [11,17].

Risk perception and preventive behaviors are the result of complicated social, cultural, and psychological factors as well as objective information [18]. This suggests that because of the differences in economic, social, and environmental contexts, residents of different regions might have different attitudes towards bottled water. In an earlier study, the findings showed that people in the Pacific region had more per capita consumption of bottled water than in other places of the U.S. [11]. In this article, the regional factor is examined and the popularity of bottled water is mapped across geographic regions.

## 2. Experimental Section

### 2.1. Hypotheses

Prior studies of bottled water consumption have identified a variety of explanatory factors for consumption behavior. However, these factors have not been considered together in one single model. For example, the regional differences found between the Pacific and the rest parts of the U.S. might be due to confounding factors such as differences in community size, local water quality problems, or water supply systems. Therefore, we propose to test these variables of interest simultaneously using a logistic regression. Hypotheses regarding use of bottled water are as follows:

H1: Perceptions of poorer groundwater and surface water quality represent higher risk in drinking water and therefore are hypothesized to be associated with higher likelihood of purchasing bottle water as a primary drinking source compared to those reporting perceptions of higher water quality. Related, perceptions that drinking water is not safe are associated with higher likelihood of purchasing bottled water for drinking as a primary water source.
H2: Based on the observations about small water supply systems, we hypothesize that small water supply (community well and rural district) users are more likely to use bottled water for drinking compared to public municipal water supply users. Community size is used as a control variable.
H3: Because of the environmental impact associated with bottled water, we test the association between environmental attitudes and bottled water use. The association between the two is hypothesized to be that the more pro-environmental views a person holds, the less likely the person frequently uses bottled water for drinking.
H4: We hypothesize a regional effect on the use of bottled water, although the specific pattern about such regional differences is not clear at this stage.

Other variables tested in the logistic model include age, education, and gender.

### 2.2. Methodology

Data used for this study were collected from a national stratified random sample mail survey about water issues conducted by Dr. Robert Mahler of University of Idaho. Our analysis used data from twenty-one states, which partially cover five out of the ten U.S. EPA water regions [19]. Data were collected 2004 through 2009 (region 8 and 9, 2004; region 7, 2006; region 6, 2008; and region 4, 2009). Sample sizes for each state were calculated based on the state population and targeted sampling error of four to six percent, with anticipation that the return rate would exceed fifty percent [20]. In each individual state, samples were either randomly selected from phone books or obtained from a professional social sciences survey company (Survey Sampling International, Norwich, Connecticut). The questionnaires were pilot tested, revised, and then mailed to sampled names and addresses. The final sample size was 5,823. Standard mail survey methods [21] were followed in all the regions and institutional review board (IRB) approval was obtained from University of Idaho Office of Research Assurance prior to the survey process. Response rates of each state ranged from 37% to 70%, with median return rates reaching the targeted 50%. The questionnaires, generally about 50 questions, varied in their content and wording due to the regions’ differing priorities. However, there were a number of core questions that all states asked. It is these questions in common that make up our data set. These core survey items asked about respondents’ perceptions of water quality, use of bottled water, water supply type, general environmental attitudes, and demographic information.

Two sources of drinking water questions were of interest in this study. The first one was “where do you primarily get your drinking water.” Possible responses to this question included: private supply (private well, river, pond, lake, *etc.*), public municipal supply, small water supply systems (including rural water district and community well), and purchase bottled water. If respondents chose “purchase bottled water” for this question, they were identified as primary users of bottled water.

The second question asked if the respondent “often use bottled water for drinking purposes.” If respondents answered “yes” to this question, they were labeled as regular users of bottled water. The above two questions were not mutually exclusive, which means that a primary bottled water user may be a regular bottled water user.

First, we tested hypotheses one, three and four on the primary bottled water users using a logistic regression model. The independent variables used in this logistic regression were as follows:

*Surface and ground water quality perceptions.* Respondents were asked to rate the surface and ground water quality in their area. Responses were coded 1 = poor, 2 = fair, 3 = very good/excellent.

*Drinking water safety.* The original question asked if the respondents felt their home drinking water is safe to drink. Response options were 0 = no, and 1 = yes.

*Environmental attitudes*. Respondents were asked to indicate where they stand on environmental issues by placing a mark on a line with numbers 1 to 10, where 1 represented preference for total natural resource use and 10 represented preference for total environmental protection.

*Community size.* Community size was measured by asking respondents to choose from the options which best described their community size, although no strict definition was given to the term “community”. Community sizes were measured with five categories. 1 was “less than 3,500 people”; 2 = “3,500 to 7,000”; 3 = “7,000 to 25,000”; 4 = “25,000 to 100,000”, and 5 was “more than 100,000.”

*Age and gender*. Age was a continuous variable measuring the ages of respondents, and gender was recorded as 0 = female and 1 = male.

*Education*. Five categories of formal education levels were provided to choose from, ranging from “less than high school” to “advanced degree.”

**Figure 1.**
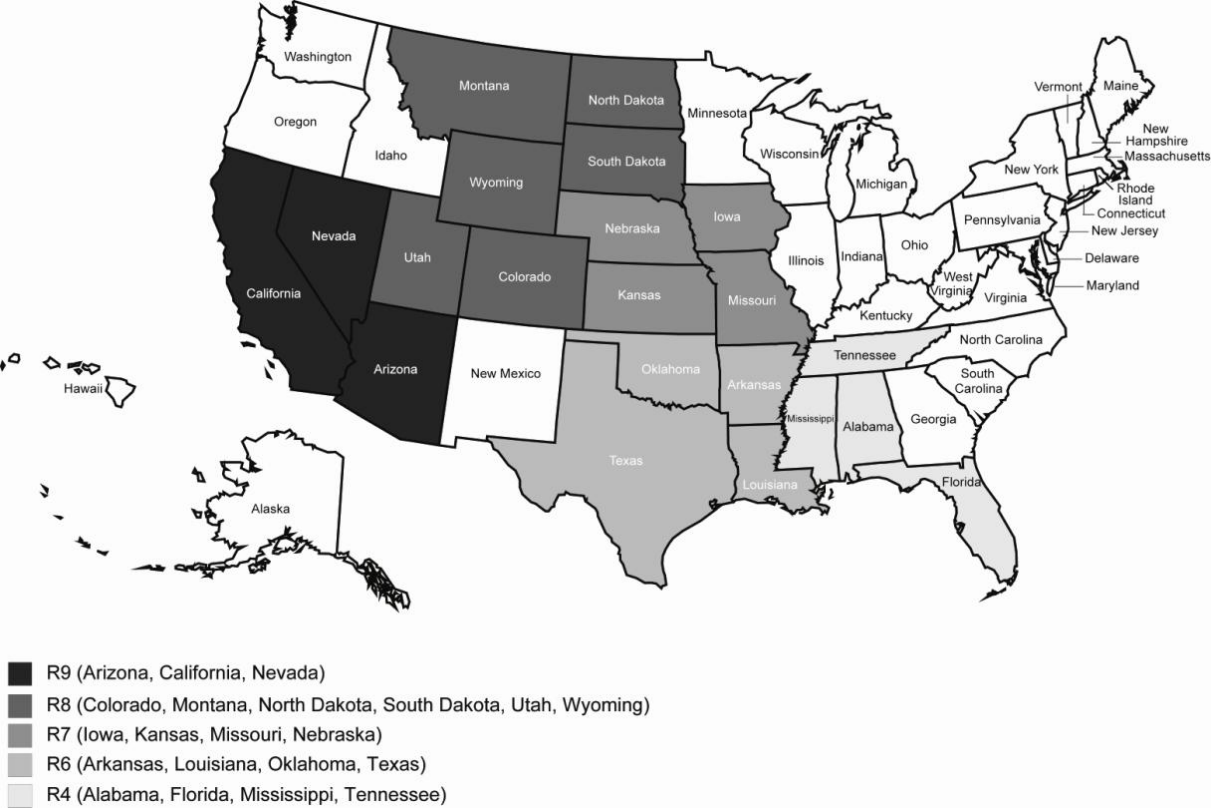
Map of the Sampled Regions and States.

*Residence region*. The two bottled water questions of interest were asked in the following regions and states, which include several states of the southeast region (Region 4: Alabama, Florida, Mississippi, Tennessee); the southern region (Region 6: Arkansas, Louisiana, Oklahoma, Texas); the Midwest Heartland region (Region 7: Iowa, Kansas, Missouri, Nebraska); the mountain region (Region 8: Colorado, Montana, North Dakota, South Dakota, Utah, Wyoming); and the southern Pacific region (Region 9: Arizona, California, Nevada [22]). Figure 1 gives a visualization of the above states and regions.

Secondly, we applied a logistic regression on the regular bottled water users. With this part of analysis, we focused on the respondents who used sources other than bottled water for primary drinking purposes but reportedly often used bottled water for drinking. The hypothesis to be tested with this model is the second one, and the independent variable of primary interest is water supply type, which has three categories: 1 = private supply (private well, river, pond, lake, *etc.*), 2 = public municipal supply, and 3 = small water supply systems (including rural water district and community well). All the other independent variables used in the previous model were also included in this logistic regression model.

## 3. Results and Discussion

### 3.1. Descriptive Summary of the Sample

The demographic distribution of survey respondents was similar to that reported for the general adult population based on the 2000 US census data for the demographic factors of community size, age (adult population), and formal education level. The only factor not in line with 2,000 census data was gender. Here, male respondents were much more heavily represented compared to the general population as a whole (about two thirds of the respondents were male, see Table 1). Even though 50% of the mailed surveys were addressed to females, it was apparent that the male adult in the surveyed household was more likely to respond to the survey [20]. The summary of sample statistics is shown in Table 1 below.

Over 13% of all respondents reported that they used bottled water as the primary source for drinking water, while 45.4% of all respondents said they often used bottled water for drinking. The mean for surface water quality perception was 1.99 (fair), and the mean for ground water quality perception was 2.22 (slightly above fair), a little higher than that of surface water. About fifteen percent respondents said they felt their home drinking water was not safe to drink. This percentage corresponded well to the percentage of respondents that used bottled water as their primary drinking source. On a scale of 1 to 10, average environmental attitude score was 5.76, and responses tended to cluster in the middle of the 1 to 10 scale. Thirty-five percent respondents marked their environmental view as 5, midway between totally eco-centric and totally anthropocentric. Other responses with higher percentage are 4 (9%), 6 (15%), and 7 (16%). About 12% respondents responded with higher scores (8–10), and the lower extreme scores (1–3) are only 6% of the total responses. This represents a balanced, somewhat more pro-environmental view towards the relationship between protection of nature and human use of natural resources. Mean age of the survey respondents was 56.8, while average formal educational achievement was between “some college” and “college degree.” About two thirds of the respondents were male.

### 3.2. Logistic Regression Model 1: Primary Bottled Water Users

Our first model used a logistic regression model to examine the relationship between primary bottled water users and water quality perceptions (Table 2).

We found that groundwater quality perception was a significant predictor. As the ground water quality perception increased by one ascending-ordered category, the odds of a person using bottled water as primary source of drinking water was reduced by 33%. Compared with a person who feels their home water is safe to drink, a person who does not trust their home drinking water safety was more than 4.8 times more likely to use bottled water as their primary source of drinking water. However, there was no significant difference in bottled water use among respondents with different surface water quality perceptions. Environmental attitudes were not a significant predictor for primary bottled water use.

Age and gender were also found to be significant predictors for bottled water use. When all other conditions were exactly equal, a respondent who was one year older in age was about 2% less likely to use bottled water as the primary source of drinking water. From a gender standpoint, the odds that a female uses bottled water for primary drinking source are 1.32 times as much as the odds for a male, with all other conditions being equal. Education level was not a significant predictor for bottled water use.

Place of residence was found to have important effect on the use of bottled water. For example, community size had a positive relationship with being a primary bottled water user. As the community size increased by one ascending category, the odds of the resident of larger community using bottled water for primary drinking purposes were increased by 0.116 times. The use of bottled water as primary source of drinking water was also closely related to where the respondents lived in the U.S. For example, a respondent in the Midwest (region 7), when compared with a respondent living in the southern Pacific region (region 9), was over 80% less likely to be a primary user of bottled water. Similarly, for a respondent in the mountain region (region 8), the odds of the person using bottled water as primary drinking water source were reduced by 53% compared with a resident in the southern Pacific region (region 9). Similar to the southern Pacific region (region 9), the southern region (region 6) and the southeast region (region 4) also have more residents primarily depending on bottled water for drinking (see appendix for detailed regional bottled water use comparison).

With logistic regression models, there is no equivalent r-squared statistics to show the explained variability in the dependent variable. However, the pseudo R^2^ shows that the explanatory variables have moderate strength of associations with consumption of bottled water. The model non-significant chi-square test and likelihood ratio test statistics (1.0), which suggests good model fit [23].

Overall, this model shows that U.S. consumer perceptions about groundwater quality have strong associations on the purchase of bottled water for drinking. This suggests that bottled water use may be considered a substitute for other water sources when groundwater quality is perceived to be poor.

### 3.3. Logistic Regression Model 2: Regular Bottled Water Users

A second logistic regression model was used to predict regular users of bottled water (Table 3).

These results show similar patterns as with primary bottled water users found in Table 2. Groundwater quality perception, safe drinking water perception, age, gender, and region of residence were found to be significant predictors. Community size, however, unlike in the first regression model, was not significant. The likelihood of private water supply users being regular bottled water users was about 25% less than that of small water supply system users. There were no significant differences in bottled water use between municipal water supply users and small water supply system users.

The pseudo r-squared statistics are relatively small compared with our first model, which suggests that the same independent variables do not have particularly strong correlations with or explaining power for regular bottled water usage, although the chi-square test statistic is still non-significant.

### 3.4. Discussion

With findings of both logistic models, we confirmed the hypothesized negative association between perception of ground water quality and bottled water use. Given that an estimate of 49% of the U.S. population depends on groundwater for its drinking water supply from either a public source or private well [24], the groundwater quality perception seems to explain the consumers’ behavior regarding bottled water. Perception of drinking water safety is found to be highly associated with bottled water use. The findings about water quality perceptions generally confirmed that when public doubts about the safety of their tap water, they look for alternatives like bottled water [6,14]. No significant relationship, however, was found between surface water quality perception and bottled water use.

Our data do not include actual water quality or safety conditions so it is not known whether consumer’s perceptions of the condition of their local drinking water are accurate reflections of the real water quality or not. If perceptions are accurate, then community leadership along with regulatory agencies needs to act to correct the problems for public health to be maintained. However, one might ask why consumers have turned to bottled water purchases rather than voice their concern and pressure public water departments and elected officials for solutions. This is particularly relevant since it is public municipal and rural water system supply users rather than private water supply users that are likely to purchase bottled water. Public water systems are tax supported, regulated and maintained under much more rigorous monitoring and testing conditions than bottled water manufacturers. This suggests that if a large number of consumers purchase drinking water as a substitute for public tap water, they can undermine the water infrastructure investments needed to assure safe public water supplies. This has implications for community capacities to provide low cost, accessible, and safe drinking water for their entire population. Without safe public water supplies, limited income households’ health and well-being are at risk.

Our findings show that although municipal water supply users and small water supply users were equally likely to be regular bottled water users when every other condition is held the same, private water supply users (private well or surface water sources) were less likely to use bottled water than small water supply users. Consumers on private wells are often targets of public health campaigns reminding them to have their water tested regularly. To the extent this happens, private water supply users may believe they have more knowledge of and control over the quality of their water supply and thus trust it. Also, media coverage and increased headlines concerning problems with public water systems around the world can lead to high distrust (appropriately) of local water supplies [14]. The poor water conditions also increase the cost of treating water in public systems so that it is safe for consumption. This can lead to changes in water taste despite being safe to drink after treatments. While substituting bottled water for public tap water under these circumstances may be a short term “fix”, it does not address long term problems of water quality or the effect it has on escalating the cost of public water as increased treatments become necessary.

Residents of larger communities were found to be more likely to be primary bottled water users, which means that a higher proportion of population in larger communities tend to depend on bottled water rather than their tap water for drinking purpose. Note that this association is established when other conditions are controlled for. That is, for two persons in the same region, with the same perceptions towards their drinking water, surface and ground water quality, and having exactly the same demographic characteristics (age, gender, education), the person from larger community is more likely to depend on bottled water for drinking purpose. As some researchers have suggested, factors like media hype about water supply problems, commercial campaigns on bottled water, or even peer pressure for more fashionable ways of drinking all contribute to bottled water consumption [6,14]. And considering that these factors are usually stronger in larger cities, it is likely that people in larger cities have more negative feelings about their water supply systems and turn to bottled water for solution. However, if respondents were already using some sort of water supply for drinking purpose, then there is no significant association found between their community size and whether or not they regularly consume bottled water. With limited information in our data we were not able to fully explain the associations found between community size and bottled water consumption, and we suggest future research look at community level variables for possible answers.

Our data also show that younger people and females are more likely to purchase bottled water. Young people are generally believed to be more susceptible to marketing and advertising, which are essential keys held by the bottled water companies [6,14]. And the higher likelihood of female drinking bottled water is consistent with previous literature on gender differences in risk, especially health and food related risk perceptions [25,26]. The findings about more consumption in these two groups of people suggests a need to target these audiences with messages about the importance of learning about their local water quality as well as the costs and quality differences between bottled water and public drinking water supplies.

Our hypothesis about environmental attitudes was not supported by the data. The relationship between environmental attitudes and bottled water use was not significant. Consumers with stronger overall concern about the environment do not seem to transfer this concern to pollution and waste problems associated with purchasing bottled drinking water. But again, because of the relatively longer cycle of research using multistate data (data collection in some states were done back in 2004), our data might not be able to reflect the newest trend of national environmental concern on bottled water.

Finally, the hypothesized regional effect regarding bottled water use was confirmed by the data. Residents of the Midwest and west mountain regions were far less likely to use bottled water for either primary drinking purpose or other occasions of regular uses, while residents of the southern pacific, the south, and the southeast were all equally likely to be bottled water users. This suggests that other variables such as culture, actual water quality conditions, media coverage of water issues and other place specific factors may be influencing the decision to use bottled water versus tap water from a private or public system. Water resource quantity and income might also be driving forces for the differences. Further research is needed to better explain regional variations.

## 4. Conclusions

Water is essential to human health and life. Access to safe water supplies and affordability are central concerns of public health and individual consumers. In this study we find that perceptions of ground water quality and local water supply safety are associated with decisions to purchase bottled water versus use public water systems for drinking water. When local water is not considered safe or of high quality U.S. consumers are more likely to use bottled water as a primary water source. Furthermore, negative perceptions of safety increase the likelihood of a consumer frequently purchasing bottled water regardless of whether their primary source of drinking water is a small water system or large municipal water supply system.

Two key implications of our findings are that (1) public health officials and community leaders need to work to assure that public municipal drinking water supplies are safe; in addition, they should find effective ways to communicate to local residents the safety of their water supply; and (2) environmental leaders and activists need to campaign about the long lasting impacts of plastic water bottles. Further the public must be engaged in understanding the relationship of water quality to the capacity of local water systems to maintain safety and good taste standards. Consumer distrust of their groundwater quality should be leveraged to create community action to address legitimate concerns.

## Figures and Tables

**Table 1.** Summary Statistics.

**Variable Name**	**Description**	**Responses**	**Mean/%**	**Std. Dev.**
Primary bottled water users	Do you primarily purchase bottled water for your drinking water? (N = 5,823)	0 = no	86.6%	
		1 = yes	13.4%	
Regular bottled water users	I often use bottled water for drinking purpose (N = 5,821)	0 = no	54.6%	
		1 = yes	45.4%	
Surface water quality	What is the quality of surface water (rivers, streams, lakes) where you live? (N = 5,142)	1 = poor	1.99	0.482
		2 = fair		
		3 = excellent		
Ground water quality	What is the quality of groundwater (sources of well water) in your area? (N = 4,166)	1 = poor	2.22	0.590
		2 = fair		
		3 = excellent		
Environmental attitudes	Indicate how you see yourself on environmental issues (N = 5,797)	1 = for total natural resource use	5.76	1.580
		10 = for total environmental protection		
Age	Age of respondents	Range from 18 to 95	56.39	16.094
Community size	What size do you consider your community to be? (N = 5,749)	1 = less than 3,500 people	3.63	1.386
		2 = 3,500 to 7,000 people		
		3 = 7,000 to 25,000 people		
		4 = 25,000 to 100,000 people		
		5 = more than 100,000 people		
Education	What level of education you have completed? (N = 5,930)	1 = less than high school or some high school	3.40	1.125
		2 = high school graduate		
		3 = some college or vocational training		
		4 = college graduate		
		5 = advanced college degree		
Gender	What is your gender? (N = 5,603)	0 = female	33.7%	
		1 = male	66.3%	
Public water supply	Where do you primarily get you drinking water? (N = 5,044)	1 = private water supply (from private well, river, pond, lake)	13.5%	
		2 = public municipal supply	73.1%	
		3 = small water systems (community well or rural water district)	13.4%	
Region	EPA water geographical regions (N = 5,823)	4 (AL, FL, MS, TN)	23.1%	
		6 (AR,LA, OK, TX)	19.3%	
		7 (IA, KS, MO, NE)	16%	
		8 (CO, MT, ND, SD, UT)	17.1%	
		9 (AZ, CA, NV)	24.6%	

**Table 2.** Logistic Regression for primary bottled water users (N = 3,232).

**Variables**	**OR (Odds Ratio)**	**CI 95%**
Surface water quality	0.778*	0.593–1.021
Ground water quality	0.670***	0.529–0.849
Environmental attitudes	0.967	0.897–1.041
Age	0.978***	0.970–0.986
Community size	1.116**	1.014–1.229
Education	0.91	0.817–1.025
Gender (control: male)	1.323**	1.025–1.707
Drinking water (control: safe)	5.884***	4.464–7.757
Region (control: region 9) Region 4 Region 6 Region 7 Region 8	0.764 0.966 0.191 *** 0.470 ***	0.550–1.061 0.692–1.348 0.108–0.337 0.317–0.697
**Pseudo R^2^ (Nagelkerke)**	**0.263**	

Note:

* P < 0.10;

** P< 0.05;

*** < 0.001.

**Table 3.** Logistic Regression for regular bottled water users (N = 2,850).

**Variables**	**OR**	**CI 95%**
Surface water quality	1.049	0.874–1.259
Ground water quality	0.661***	0.566–0.771
Environmental attitudes	0.973	0.925–1.024
Age	0.984***	0.979–0.989
Community size	1.046	0.981–1.116
Education	1.039	0.965–1.120
Gender (control: male)	1.243**	1.035–1.493
Drinking water (control: safe)	1.742***	1.307–2.323
Region (control: region 9) Region 4 Region 6 Region 7 Region 8	0.988 1.144 0.541 *** 0.528 ***	0.785–1.243 0.896–1.461 0.413–0.708 0.413–0.676
Small water supply systems Private water supply Municipal water supply	1.746** 0.896	0.560–0.993 0.709–1.132
**Pseudo R^2^ (Nagelkerke)**	**0.094**	

Note:

* P < 0.10;

** P < 0.05;

*** P < 0.001.

## Appendix 1

A separate analysis, a one-way ANOVA (analysis of variance) was done to compare regional differences in bottled water use for primary drinking purposes. Table 1 shows bottled water use in each region and differences with statistical significance. The variable (primarily purchase bottled water for drinking) is a dichotomous variable with two possible responses 0 (not purchase) and 1 (purchase bottled water for drinking). Therefore the following means reflect proportion of respondents responding with 1 in each region. Post-hoc Bonferroni pair tests were conducted on the means and the last column of the following table shows regions with significant differences (at 0.05 level). For example, the first row shows that region 4 has mean which is significantly different from that of region 6, 7, 8, and 9, respectively.

**Table 1.** Bottled water use by region.

Region	N	Mean	S.D.	Regions with significant differences
R4	1,344	0.136	0.343	R6, R7, R8, R9
R6	1,122	0.185	0.389	R4, R7, R8
R7	932	0.045	0.208	R4, R6, R9
R8	993	0.063	0.244	R4, R6, R9
R9	1,432	0.198	0.398	R4, R7, R8

Region 9 and region 6 have significantly higher percent of primary bottled water users, followed by region 4. Region 7 and region 8 have the least primary bottled water users.
